# Larger preoperative medial meniscus extrusion is associated with insufficient pain relief following pullout repair for medial meniscus posterior root tears

**DOI:** 10.1002/jeo2.70822

**Published:** 2026-06-24

**Authors:** Koki Kawada, Takayuki Furumatsu, Yuki Okazaki, Toshiki Kohara, Toshifumi Ozaki

**Affiliations:** ^1^ Department of Orthopaedic Surgery Okayama University Graduate School of Medicine, Dentistry, and Pharmaceutical Sciences Okayama Japan; ^2^ Department of Orthopaedic Surgery Japanese Red Cross Okayama Hospital Okayama Japan

**Keywords:** medial meniscus extrusion, meniscus, posterior root tear, postoperative pain, pullout repair

## Abstract

**Purpose:**

We aimed to evaluate preoperative factors associated with postoperative pain after transtibial pullout repair for medial meniscus posterior root tears (MMPRT), focusing on patient characteristics and imaging findings, including bone morphology and preoperative medial meniscus extrusion (MME).

**Methods:**

Patients who underwent isolated transtibial pullout repair for MMPRT at our institution were included in this retrospective study. Preoperative knee joint radiographs were evaluated for the Kellgren–Lawrence grade and bone morphology. Additionally, MME was measured using preoperative and 1‐year postoperative magnetic resonance imaging. The Knee Injury and Osteoarthritis Outcome Scores (KOOS)‐Pain subscale was evaluated preoperatively and at final follow‐up. Univariate and multivariate linear regression analyses were conducted to evaluate the association between postoperative KOOS‐Pain and preoperative factors. Additionally, surgical revision rates for around‐knee osteotomy and arthroplasty were evaluated.

**Results:**

Statistical analyses were conducted for 508 knees, of which 406 (79.9%) were female. The mean age was 65.3 ± 9.1 years (range, 24–86), and mean body mass index was 25.7 ± 4.2 kg/m^2^ (range, 15.6–49.4). The mean final follow‐up period was 25.0 ± 17.2 months (range, 12–72). Sex (*p* = 0.028), preoperative MME (*p* = 0.016), and preoperative KOOS‐Pain (*p* < 0.001) in univariate linear regression analyses were significantly associated with postoperative KOOS‐Pain. Subsequently, in the multivariate linear regression model, preoperative MME (*p* = 0.024) and preoperative KOOS‐Pain (*p* < 0.001) were independent predictors of postoperative KOOS‐Pain. No patients underwent conversion to around‐knee osteotomy or arthroplasty occurred during follow‐up period.

**Conclusions:**

In a large cohort of 508 knees, preoperative MME was independently associated with postoperative KOOS‐Pain after transtibial pullout repair for MMPRT with early‐stage knee osteoarthritis (Kellgren–Lawrence grade ≤ 2). The degree of preoperative MME does not predict failure of pullout repair for MMPRT; however, it is important to understand its association with postoperative pain and consider the indications for surgical intervention.

**Level of Evidence:**

Level IV.

**Abbreviations**
ADL: activities of daily living
BMI: body mass index
ICC: intraclass correlation coefficients
IKDC: International Knee Documentation Committee
KOOS: Knee Injury and Osteoarthritis Outcome Score
MME: medial meniscus extrusion
MMPR: medial meniscus posterior root
MMPRT: medial meniscus posterior root tears
MRI: magnetic resonance imaging
OA: osteoarthritis
SIFK: subchondral insufficiency fracture of the knee

## INTRODUCTION

Medial meniscus posterior root (MMPR) tears (MMPRT) have gained widespread recognition, and a robust association with knee osteoarthritis (OA) has been shown [2]. The importance of early diagnosis and proper management of MMPRT has been increasingly emphasized [2, 3]. Furthermore, poor clinical outcomes with conservative treatment for MMPRT have been reported, and early surgical repair is being recommended more frequently [3, 23]. Conversely, MMPR repair has reportedly yielded relatively satisfactory mid‐term to long‐term outcomes with a low rate of knee arthroplasty conversion [4, 15, 28]. However, knee OA progression cannot be completely prevented even after MMPR repair, with some cases requiring a conversion to knee arthroplasty due to repair failure or persistent knee pain [15, 18].

Regarding clinical scores after MMPR repair, a previous report indicated that clinical outcomes improved over time during the 3‐year postoperative period [20]. In contrast, reports show minimal clinically important differences and patient acceptable symptom state achievement rates at 2 years following transtibial pullout repair, an arthroscopic surgical procedure used to fix meniscal root tears, for MMPRT of approximately 60%, indicating that some patients experience persistent symptoms [13]. Previous reports have identified several factors associated with poor postoperative outcomes after MMPR repair, including advanced age, high body mass index (BMI), lower limb varus deformity >5°, severe cartilage lesions and postoperative medial meniscus extrusion (MME) progression [6, 7, 16, 31, 36].

However, the relationship between preoperative MME and postoperative clinical outcomes remains controversial. Dzidzishvili et al. reported that a preoperative MME > 3 mm predicted poor postoperative clinical scores [11]. Conversely, some reports indicate that a preoperative MME > 3 mm does not correlate with postoperative clinical scores [7, 35]. Therefore, in this study, we aimed to evaluate the preoperative factors associated with postoperative pain after MMPR repair, focusing on patient characteristics and imaging findings, including bone morphology and preoperative MME. The hypothesis of this study was that a greater preoperative MME would predict persistent knee pain during activities of daily living (ADL) after MMPR repair.

## METHODS

### Patients

This retrospective study was conducted in accordance with the Declaration of Helsinki and was approved by our institutional review board. All the participants provided written informed consent at their initial visit for the use of anonymized patient data and clinical scores in research.

**Figure 1:**
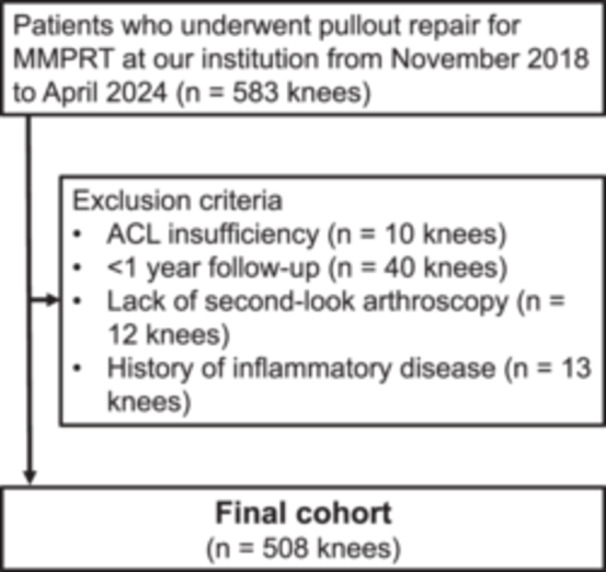
Flowchart of the study. ACL, anterior cruciate ligament; MMPRT, medial meniscus posterior root tears.

Patients who underwent isolated transtibial pullout repair for MMPRT at our institution between November 2018 and April 2024 were included in this study (Figure 1). All surgeries were performed by a single surgeon. Patients were excluded if they had a history of anterior cruciate ligament insufficiency or injury, < 1 year of follow‐up, or a history of inflammatory disease. Furthermore, since most of the included patients underwent second‐look arthroscopy, those who did not were also excluded from the final cohort to standardize baseline characteristics.

### Surgical indication, technique and rehabilitation protocol

At our institution, the indications for repair of MMPRT include symptomatic knee pain, absence of severe varus alignment (femorotibial angle > 180°) [17], absence of advanced knee OA (Kellgren–Lawrence grade ≥ 3) [31], and absence of severe cartilage lesions (International Cartilage Repair Society grade ≥ 3). Contraindications for MMPRT repair based on age, BMI, or time from injury to surgery were not considered.

All the patients underwent transtibial pull‐out repair. MMPRT classification was conducted based on a report by LaPrade et al. [24]. Two sutures were applied to the MMPR ends using two simple or two cinch stitches. The sutures were then pulled into a 4.0–4.5 mm tibial bone tunnel created very close to the anatomical MMPR attachment. Finally, suture fixation was achieved using a 5.0‐mm Biosure RG (Smith & Nephew) and cortical screw as an anchor screw on the anterior aspect of the tibia.

The standard postoperative rehabilitation protocol included knee immobilization with a brace and non‐weight‐bearing for the first postoperative week. Knee range of motion was subsequently increased by 30° per week, and weight bearing was progressively advanced by 20 kg per week. Knee flexion was restricted to a maximum of 120° during the first 2 months postoperatively. Patients were allowed to resume sports activities 3 months after surgery.

### Radiographic and magnetic resonance imaging (MRI) assessment

For radiographic evaluation, weight‐bearing anteroposterior and non‐weight‐bearing lateral radiographs of the knee were obtained preoperatively. The Kellgren–Lawrence grade, femorotibial angle, medial proximal tibial angle and joint line convergence angle were assessed following the weight‐bearing anteroposterior radiographs [17, 26, 30]. Additionally, the medial posterior tibial slope was measured using non‐weight‐bearing lateral radiographs. The radiographic images of the lateral knee joint were unified so that the posterior condyles of the femur overlapped [35].

**Figure 2:**
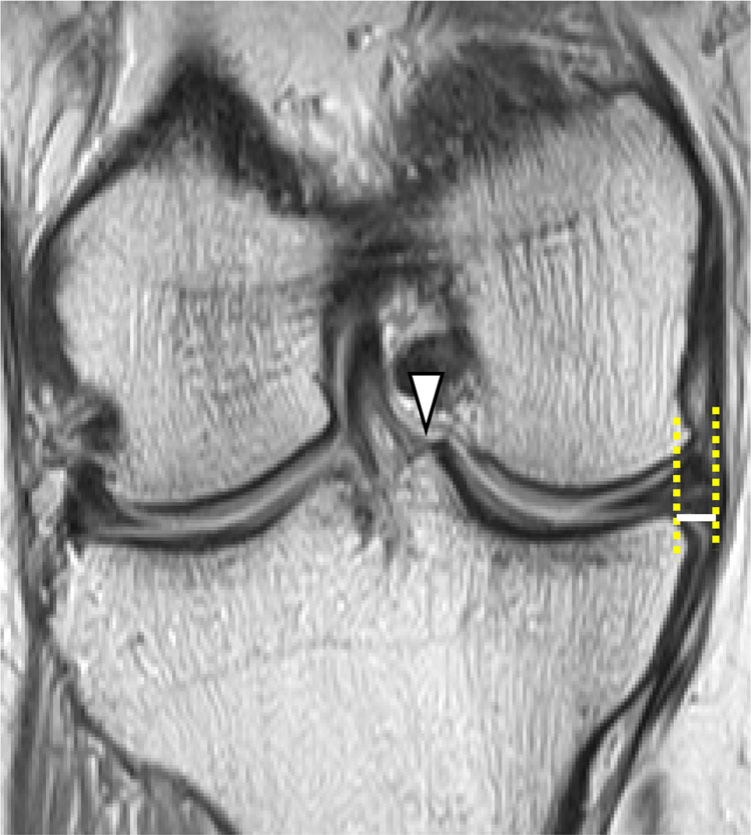
MME measurement. MME (white line) was defined as the distance from the medial margin of the tibial plateau, excluding osteophytes, to the medial margin of the medial meniscus on the coronal slice (yellow dotted lines), where the medial tibial eminence was the highest (white arrowhead). MME, medial meniscus extrusion.

Pre‐ and post‐operative MME for the MRI evaluation in the non‐weight‐bearing position was measured on coronal images. MME was defined as the distance from the medial margin of the tibial plateau, excluding osteophytes, to the medial margin of the medial meniscus on the coronal slice, where the medial tibial eminence was the highest (Figure 2) [18].

### Pain score assessment

Clinical score was assessed preoperatively and at the final follow‐up. Pain was evaluated using the Knee Injury and Osteoarthritis Outcome Score (KOOS)–Pain subscale, which is a patient‐reported and self‐administered outcome measure [28]. Scores range from 0 to 100, with higher scores indicating less pain.

### Conversion to around ‐ knee osteotomy or arthroplasty

The incidence of subsequent surgical conversion was evaluated. Conversion was defined as any additional surgical procedure involving around‐knee osteotomy or arthroplasty performed after pullout repair for MMPRT during the follow‐up period.

### Statistical analysis

Statistical analyses were conducted using the EZR software (Saitama Medical Center). The normality of each variable was assessed before analysis. A *p* value < 0.05 was considered statistically significant.

The KOOS‐Pain and MME were compared pre‐ and post‐operatively using the Wilcoxon signed‐rank test.

Univariate linear regression analyses were conducted to evaluate the association between postoperative KOOS‐Pain and preoperative factors. Variables with a *p* value < 0.200 in the univariate analyses were subsequently entered into a multivariable linear regression model to identify independent predictors of postoperative KOOS‐Pain.

Two orthopaedic surgeons specializing in knee joints independently measured radiographic and MRI assessment. One observer repeated the measurements to assess intra‐observer reliability. Inter‐ and intra‐observer reliabilities were evaluated using intraclass correlation coefficients (ICC). ICC values were interpreted as follows: <0.50, poor; 0.50–0.75, moderate; 0.75–0.90, good; and >0.90, excellent reliability. Model fit was evaluated using the coefficient of determination (*R* ^2^) and adjusted *R* ^2^. A post hoc power analysis was performed using G*Power 3.1 (Heinrich Heine University) based on the observed effect size of the final multivariable model (*F* test for linear multiple regression, fixed model, *R* ^2^ deviation from zero).

## RESULTS

Initially, a total of 583 knees in patients who underwent isolated transtibial pullout repair for MMPRT at our institution between November 2018 and April 2024 were included (Figure 1). Patients with a history of anterior cruciate ligament insufficiency or injury (*n* = 10), <1 year of follow‐up (*n* = 40), absence of second‐look arthroscopy (*n* = 12), or a history of inflammatory disease (*n* = 13) were excluded. Ultimately, 508 knees were included in the statistical analysis.

Table 1 shows patient characteristics and preoperative radiographic and MRI findings. The mean age was 65.3 ± 9.1 years (range, 24–86), and 406 knees (79.9%) were female. The mean BMI was 25.7 ± 4.2 kg/m^2^ (range, 15.6–49.4). The mean final follow‐up period was 25.0 ± 17.2 months (range, 12–72), with a median follow‐up of 12 months. The mean MME increased significantly from 4.5 ± 1.2 mm preoperatively to 5.9 ± 1.6 mm at 1 year postoperatively (*p* < 0.001). KOOS‐Pain showed significant improvement from preoperative (59.0 ± 17.4) to final evaluation (87.2 ± 12.0; *p* < 0.001).

**Table 1:** Patient characteristics.

Characteristics	Values	Range
Patients, knees	508	
Age (years)	65.3 ± 9.1	24–86
Sex, male/female, knees (%)	102 (20.1)/406 (79.9)	
Body mass index (kg/m^2^)	25.7 ± 4.2	15.6–49.4
Preoperative KL grade, 0/1/2/3/4, knees	1/199/308/0/0	
Preoperative FTA (°)	178.2 ± 1.7	172–180
Preoperative MPTA (°)	84.9 ± 1.8	80–93
Preoperative MPTS (°)	10.0 ± 3.1	2–19
Preoperative JLCA (°)	1.3 ± 1.3	−2 to 5
Preoperative MME (mm)	4.5 ± 1.2	1.6–8.8
MMPRT classification, 1/2/3/4/5, knees	55/436/0/17/0	
Follow‐up period (months)	25.0 ± 17.2	12–72

*Note*: Values are presented as the means ± standard deviation or numbers.

Abbreviations: FTA, femorotibial angle; JLCA, joint line convergence angle; KL, Kellgren‐Lawrence; MME, medial meniscus extrusion; MMPRT, medial meniscus posterior root tears; MPTA, medial proximal tibial angle; MPTS, medial posterior tibial slope.

Sex (*p* = 0.028), preoperative MME (*p* = 0.016) and preoperative KOOS‐Pain (*p* < 0.001) in univariate linear regression analyses were significantly associated with postoperative KOOS‐Pain (Table 2). Preoperative MME (*p* = 0.024) and KOOS‐Pain score (*p *< 0.001) in the multivariate linear regression model remained independent predictors of postoperative KOOS‐Pain (Table 3).

**Table 2:** Univariate linear regression analyses between postoperative KOOS‐Pain and　preoperative factors.

Preoperative factor	*β* coefficient	95% CI	*p* Value
Age (years)	−0.013	−0.128 to 0.101	*p* = 0.818
BMI (kg/m^2^)	−0.189	−0.439 to 0.060	*p* = 0.139
Gender (male vs female)	2.924	0.325–5.523	***p*** = **0.028** *
Preoperative FTA (°)	−0.467	−1.093 to 0.158	*p* = 0.143
Preoperative MPTA (°)	0.248	−0.318 to 0.814	*p* = 0.390
Preoperative MPTS (°)	0.090	−0.250 to 0.430	*p* = 0.604
Preoperative JLCA (°)	−0.307	−1.093 to 0.479	*p* = 0.444
Preoperative MME (mm)	−1.093	−1.985 to −0.201	***p*** = **0.016** *
Preoperative KOOS‐Pain	0.246	0.190–0.302	***p*** < **0.001** *

*Note*: Bold values indicate statistical significance.

Abbreviations: BMI, body mass index; CI, confidence interval; FTA, femorotibial angle; JLCA, joint line convergence angle; KOOS, Knee Injury and Osteoarthritis Outcome Score; MME, medial meniscus extrusion; MPTA, medial proximal tibial angle; MPTS, medial posterior tibial slope.

* *p* < 0.05.

**Table 3:** Multivariable linear regression analysis between postoperative KOOS‐Pain and preoperative factors.

Preoperative factor	*β* coefficient	95% CI	*p* Value
BMI (kg/m^2^)	−0.148	−0.385 to 0.092	*p* = 0.227
Gender (male vs female)	1.639	−0.881 to 4.158	*p* = 0.202
Preoperative FTA (°)	−0.413	−1.002 to 0.176	*p* = 0.169
Preoperative MME (mm)	−0.966	−1.803 to −0.129	***p*** = **0.024** *
Preoperative KOOS‐Pain	0.236	0.179–0.293	***p*** < **0.001** *

*Note*: Bold values indicate statistical significance.

Abbreviations: BMI, body mass index; CI, confidence interval; FTA, femorotibial angle; KOOS, Knee Injury and Osteoarthritis Outcome Score; MME, medial meniscal extrusion.

* *p* < 0.05.

No patients (0 of 508 knees) underwent conversion to osteotomy or arthroplasty during follow‐up period.

All radiographic and MRI parameters demonstrated good to excellent reliability.　The ICC values for intra‐ and inter‐observer reliability ranged from 0.84 to 0.95 and 0.83 to 0.94, respectively. MME measurement showed excellent reproducibility (intra‐observer ICC = 0.95; inter‐observer ICC = 0.94). The post hoc power analysis performed using G*Power demonstrated that the multivariable regression model had sufficient statistical power to detect the observed effect size (*f* ^2 ^= 0.17) with the available sample size (*N* = 508, *α* = 0.05, five predictors). The achieved power was greater than 0.99.

## DISCUSSION

The most important finding of this study was that preoperative MME was independently associated with postoperative pain during ADL, as assessed by postoperative KOOS‐Pain.

In MMPRT treatment, greater MME and advanced age are also known risk factors for knee OA progression during conservative treatment [21]. Furthermore, MME progression over time leads to the failure of conservative treatment for MMPRT and increases the need for transtibial pullout repair [19]. However, the effect of preoperative MME on postoperative outcomes remains controversial [7, 11, 35]. Therefore, within the scope of previous reports, a large preoperative MME in patients undergoing MMPRT is considered to indicate the limitations of conservative treatment, rather than those of repair surgery. Previous studies have reported correlations between preoperative MME and the tear gap of the MMPRT [14], between the tear gap of the MMPRT and postoperative clinical outcomes [33], between preoperative MME and subchondral insufficiency fracture of the knee (SIFK) [8], and between SIFK and postoperative clinical outcomes [12]. This finding indicated an indirect association between preoperative MME and postoperative clinical outcomes. In this study, preoperative MME was an independent factor associated with postoperative KOOS‐Pain. However, regardless of the degree of postoperative pain, no patients required conversion to around‐knee osteotomy or arthroplasty during the follow‐up period. Thus, the results of this study do not necessarily imply that patients with a large preoperative MME should be excluded from being a candidate for transtibial pullout repair for MMPRT. Nevertheless, persistent postoperative pain may eventually lead to conversion to osteotomy or arthroplasty over longer‐term follow‐up. Therefore, our findings highlight that for patients with a larger preoperative MME, careful consideration is required when determining whether repair surgery is appropriate.

The degree of MME correlates with increased joint loading in biomechanical studies [10]. Also, postoperative MME progression was associated with mid‐term to long‐term clinical scores [26]. Therefore, preventing postoperative MME progression has become a key outcome of MMPR repair. However, the MME cannot be fully restored even after MMPR repair [34]. In this study, MME also showed significant progression at 1 year postoperatively compared to preoperative levels. Procedures such as meniscal centralization have been proposed as methods to reduce MME [30]. However, the effectiveness of centralization as an isolated repair technique remains limited [34], and further refinement of surgical strategies are required.

In addition to MME, factors such as advanced age, high BMI, lower limb varus alignment >5° and severe cartilage lesions have been reported as preoperative factors associated with postoperative clinical outcomes [6, 7, 16, 31, 36]. Patients with excessive lower limb varus alignment or severe cartilage lesions were excluded as candidates for MMPR repair in this study. Under these conditions, advanced age and a high BMI were not significantly associated with postoperative KOOS‐Pain. Meanwhile, in this study, preoperative KOOS‐Pain was an independent predictor of postoperative KOOS‐Pain, along with preoperative MME. To date, no reports have indicated that preoperative pain during MMPR repair correlates with postoperative pain. However, several reports on knee surgeries other than MMPR repair have suggested that preoperative pain associated with psychological factors, including preoperative catastrophizing, may be associated with persistent postoperative knee pain [1, 22]. In this study the significance of preoperative pain could not be fully evaluated because the psychological effects were not assessed. Previously, the association between the duration of the pain period and pain catastrophizing has been reported [5, 9]. This indicates that in MMPRT treatment strategies, earlier diagnosis and treatment could potentially enhance postoperative pain management.

However, this study had some limitations. First, it was a retrospective study. Second, the absence of a control group prevented a direct comparison with conservative or other treatments. Third, a single surgeon conducted all surgeries, requiring caution when generalizing the results. Fourth, the MRI was conducted in a non‐weight‐bearing position, potentially limiting its ability to fully reflect meniscal dynamics under weight‐bearing conditions. Fifth, psychological factors, such as pain catastrophizing, were not evaluated in this study, potentially limiting the assessment of the relationship between pre‐ and post‐operative pain. Sixth, the pullout repair indications for MMPRT in our institution were limited to patients with early‐stage knee OA (Kellgren–Lawrence grade ≤ 2), thereby limiting generalizability of the study. Seventh, the follow‐up period was relatively short, averaging 2 years (12–72 months), and for assessing joint survival rates, longer‐term evaluation is necessary. Prospective, multicenter studies with prolonged follow‐up are needed to validate these findings and to further clarify long‐term clinical outcomes, including qualitative aspects of postoperative pain.

## CONCLUSIONS

In a large cohort of 508 knees, preoperative MME was independently associated with postoperative KOOS‐Pain after MMPR repair. The degree of preoperative MME does not predict failure of pullout repair for MMPRT; however, it is important to understand its association with postoperative pain and consider the indications for surgical intervention.

## AUTHOR CONTRIBUTIONS

*Conceptualization*: Takayuki Furumatsu and Koki Kawada. *Methodology*: Takayuki Furumatsu and Koki Kawada. *Formal analysis and investigation*: Koki Kawada and Toshiki Kohara. *Writing—original draft preparation*: Takayuki Furumatsu and Koki Kawada. *Writing—review and editing*: Takayuki Furumatsu, Koki Kawada, Yuki Okazaki, Toshiki Kohara and Toshifumi Ozaki. *Supervision*: Toshifumi Ozaki.

## CONFLICT OF INTEREST STATEMENT

The authors declare no conflicts of interest.

## ETHICS STATEMENT

This study was performed in accordance with the principles of the Declaration of Helsinki. Approval for carrying out the research work was granted by the Ethics Committee of the Okayama University (No. 1857). Written informed consent was obtained from all the patients.

## Data Availability

The data that support the findings of this study are available from the corresponding author, upon reasonable request. The data are not publicly available due to privacy or ethical restrictions.
